# Editorial: Natural products and brain energy metabolism: Astrocytes in neurodegenerative diseases Volume II

**DOI:** 10.3389/fphar.2023.1137554

**Published:** 2023-01-17

**Authors:** Fushun Wang, Shijun Xu, Fang Pan, Alex Verkhratsky, Jason H. Huang

**Affiliations:** ^1^ Institute of Brain and Psychological Science, Sichuan Normal University, Chengdu, China; ^2^ School of Pharmacy, Chengdu University of Traditional Chinese Medicine, Chengdu, China; ^3^ Department of Medical Psychology, Shandong University Medical School, Jinan, China; ^4^ Department of Physiology, The University of Manchester, Manchester, United Kingdom; ^5^ Department of Neurosurgery, Baylor Scott & White Health, Temple, TX, United States; ^6^ Department of Surgery, Texas A&M University College of Medicine, Temple, TX, United States

**Keywords:** energy supply, astrocyte, neurodegenerative (Alzheimer’s, emotional arousal, basic emotions, three primary color

## Introduction

The energy supply is critically important for the normal function of the brain, because the brain utilizes approximately 20% of the total oxygen and energy supply in the body with only 2% of body weight. In addition, the brain cannot reserve energy, thus it will get permanent damage if there is no blood supply for 5 minutes. Indeed, many studies have demonstrated that energy supply shortage might be the major causes for brain aging and neurodegenerative diseases, such as Alzheimer’s diseases. However, even though it is pivotally important to understand the physiology and pathophysiology of energy supply for the brain, the mechanisms in the regulatory processes are far from clear.

## Neuromodulators regulate brain energy supply

**FIGURE 1:**
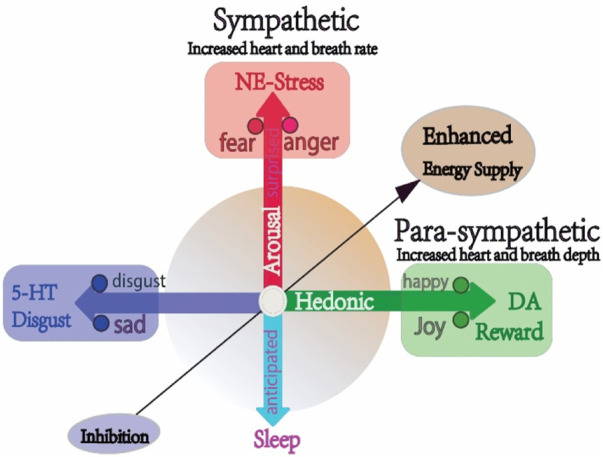
The three monoamines and three primary emotions and energy supply.

Numerous studies have demonstrated that the blood supply is modulated by brain activity induced neuromodulator release (Wang et al., 2013). The neuromodulators are the major substrates for the emotional states (Gu, et al., 2019a), for example, LC-NE (Locus Coeruleus and norepinephrine) is the major substrate for emotional arousal, while dopamine is for hedonic valence (Figure 1). Emotional arousal can activate blood supply by increasing heart rate and breath rate to supply glucose and oxygen for the brain, *via* enhancing LC-NE activity to induce “fight or flight” behaviors or fear and anger emotions. The emotional arousal and hedonic valence are two emotional dimensions (Figure 1). Basic emotional theory is another prevalent theory coming together with dimensional emotion theory, which suggested that there are a limit number of basic emotions, and each basic emotion has distinct neural and physiological activities. Even though most psychologists agree upon the theory of basic emotion, they cannot agree upon the number of basic emotions, or agree upon the links among the basic emotions (Gu, et al., 2019a). We are the first to hypothesize that there might be three primary emotions: joy, disgust and fear (Figure 1) (Gu, et al., 2018), which can be combined to form many other emotions like the three primary colors (Liang, et al., 2021). We also integrated the three primary emotion with emotional dimensions, and hypothesized that the joy represents the left pole of valence, while the disgust represents the right pole of valence, and the fear (together with anger) represents the vertical pole of dimension (arousal) (Figure 1) (Gu et al., 2019b; Jiang et al., 2022; Dong et al., 2022).

The two dimensions represent two features of emotion: arousal and valence*.* Different locations of emotions on the dimension mean that different emotions have different amounts of *arousal or hedonic parameters*. In addition, we hypothesized that there are three primary emotions, which are subsided by three monoamines: DA-joy-reward; NE-fear-anger-stress; 5-HT-disgust-sadness-punishment (Zheng et al, 2016). The activity of LC-NE and sympathetic system can increase the heart rate and energy supply; while para-sympathetic system increases the breath depth and heart pump strength. Thus both sympathetic and parasympathetic systems enhance energy supply, while the 5-HT induced sleepy states can block energy supply (adopted from our previous paper, Gu et al., 2019a; Gu et al., 2019b; 2022).

The neuromodulators can not only modulate the physiological process by increasing blood flow and heart rates, they can also affect energy supply in the central nervous system (Beley et al., 1991). For example, it is suggested that LC-NE can activate the astrocytic Ca^2+^ signaling and enhance Na^+^, K^+^-ATPase activity in astrocytes (Ding et al., 2013). Indeed, the effects of monoamine neuromodulators on the brain energy states have been thoroughly investigated by many scientists. And lacking these monoamines has been suggested to be the major reason for depression in the brain (He et al., 2021).

## Astrocytes in energy supply for the brain

The brain cannot reserve energy or reserve glucose either. The glucose is the major energy source in the brain, however, glucose can only be saved in astrocyte as glycogen. The glycogenetic hypothesis suggested that the astrocyte can reserve glucose as glycogen and release glucose at high energy demands, such as neuromodulator release (Petit et al., 2021). Consistent with norepinephrine (NE) inducing glycogen release in the livers or muscle, the neuromodulators can also induce glucose release from astrocytes. The neuromodulators have been proved to be glycogenolytic agents (DiNuzzo et al., 2015), which might work *via* Ca^2+^ signaling in astrocytes (Ding, et al., 2013). In addition, accumulating evidence indicates that emotional arousal can activate astrocytic Ca^2+^ signaling to induce glucose release (Ding et al., 2016). Consistently we reported that NE can induce astrocytic Ca^2+^ signaling and activate Na^+^ pump to actively buffer extracellular K^+^ (Wang et al., 2012). In addition, the mitochondria, whose major function is making ATP *via* glucose oxygenations, can also be modulated by NE as well as Ca^2+^ signaling (Iwata et al., 2019). Indeed, mitochondrial abnormality induced energy supply deficit might be the major cause for ageing and neurodegenerative disorders such as Alzheimer’s disease, Parkinson disease, stress, and depression etc.

## Natural product for energy supply

The blood flow or energy supply can be modulated by many nature products and the Traditional Chinese Medicine have reported many natural plants to activate the energy supply for the brain, such as Ginseng. And recently, the active ingredients in these nature plants have been abstracted, such as alkaloids, polyphenols, and flavonoids. These active compounds from plants have been examined for toxicity and for efficacy and have been examined *in vivo* and also in clinics. For example, Huperzine A from Huperzia serrata has been proven to activated the astrocytic Ca signaling and prevent the cognitive deficit in Alzheimer’s disease, and huperzine A-derived Shiplin is currently been tested in Phase III clinical studies. These studies could lead to many new drug discoveries for energy supply for treating neurodegenerative diseases. Thus we started this Research Topic to invite new discoveries in finding nature products to enhance energy supply for the brain.

## This research topic collected papers

In this Research Topic, we invited high-quality studies about the physiological and pathological mechanisms for energy supply and also in finding natural products to enhance energy metabolism at neurodegenerative diseases. We got seven article accepted *via* peer-reviewed processes, which are shown below:

In the review paper Wang et al., introduced several other kinds of Chinese herbs, including Chaihu-Shugan-San, Danggui Shaoyao San Xiaoyaosan. They reviewed recent studies about their effects on monoamine neurotransmitters, as well as on the stress hormones in hypothalamus-pituitary-adrenaline axis.

In another review paper, the authors Shi et al. reviewed recent studies about Ginsenosides, which is a major compound in ginseng. The authors suggested that ginsenoside can be effective in treating AD *via* reducing β-amyloid (Aβ) and neurofibrillary tangles through enhancing energy supply.

In the experimental paper titled the author Yi et al. introduced one kind of Chinese herb Xuefu Zhuyu decoction. The authors suggested that this kind of Chinese herb can be used in the treatment of ischemic stroke by maintaining normal glymphatic system function *via* protection of AQP4 expression and polarization.

The authors Tao et al. contributed one review paper. In the article, the authors introduced recent studies about traditional Chinese medicines for antagonizing dementia, and found that these drugs are potential new drugs for dementia with many advantages, such as few adverse effects, lower cost, but efficient effects.

In the article the authors Chen et al. suggested that physical activities and exercises can affect astrocytes in mice by changing the expression of mRNAs and corresponding proteins, and thus metabolism.

In the experimental paper, Fang et al. studied the effects of dehydrocorydaline on NLRP3 inflammasome-mediated astrocyte activation and their effects on stress induced neurotransmitter release (DA and 5-HT), possibly *via* inflammatory factors.

In another review article, Nizamutdinov et al. studied the effects of near-infrared light on brain energy metabolism *via* anti-inflammatory, detoxification, neuroprotection etc. They suggested that the near-infrared light can affect glymphatic and brain lymphatics system, which might be a good way to treat neurodegenerative diseases.

In all, this Research Topic has successfully invited seven article about the effects of natural products on the energy supply and neuromodulator release, which might be potential new therapies for neurodegenerative disorders. We hope this Research Topic will shed new lights on developing new ways for neurodegenerative disorders.
